# Herpes Zoster: A Case Report of a Rare Ramification Leading to Secondary Infection

**DOI:** 10.7759/cureus.36732

**Published:** 2023-03-27

**Authors:** Aravind Warrier S, Sivaswarubini Ganesh, Thamizhchelvan Harikrishnan, Barath Balaji, Divyambika C Venugopal, Sushmitha S

**Affiliations:** 1 Oral Medicine and Radiology, Sri Ramachandra Institute of Higher Education and Research, Chennai, IND; 2 Oral and Maxillofacial Pathology, Sri Ramachandra Institute of Higher Education and Research, Chennai, IND

**Keywords:** post-herpetic neuralgia, bacterial superinfection, actinomycotic osteomyelitis, shingles, herpes zoster

## Abstract

The herpes virus causes herpes zoster (HZ) (shingles). It develops years later in elderly patients who were affected by the varicella-zoster virus in their childhood. The virus gets reactivated and typically localizes its symptoms to a particular dermatome. If left untreated, it can lead to dental complications, such as osteonecrosis, tooth exfoliation, periodontitis, calcified and devitalized pulps, periapical lesions, and root resorption, in addition to developmental irregularities, such as abnormally short roots and missing teeth. Here, we present the case of a 61-year-old male affected by a rare bacterial superinfection followed by an HZ infection. Our report aims at making clinicians aware of the various potential complications that can develop after an HZ infection.

## Introduction

Herpes zoster (HZ) is caused by the reactivation of the latent varicella-zoster virus, which remains latent in the cranial or sensory ganglia after the primary infection in association with immunosuppression and mechanical or psychological stress leading to secondary infection, which manifests as vesicular rash and radicular pain in the affected dermatomal area [1,2]. It affects 20-30% of the population and produces severe prodromal symptoms such as eye discomfort, ocular abnormalities, or skin rash in specific dermatomes [2].

HZ can affect any of the three branches of the trigeminal nerve. The involvement of the mandibular and maxillary branches without the involvement of the ophthalmic branch is relatively rare and accounts for only 1.7% of HZ cases [3]. Oral manifestations of HZ appear when the second and third divisions of the trigeminal nerve are affected [3]. These are often self-limiting infections, and if the patient exhibits significant pain symptoms, antiviral therapy is administered both systemically and topically depending on the intensity of the pain [4].

The most common post-zoster-related complications include ocular complications, facial palsy, post-herpetic neuralgia (PHN), bacterial superinfections, osteonecrosis, periodontitis, exfoliation of teeth, calcified and devitalized pulps, periapical lesions, and root resorption [3,4]. Here, we report a case of actinomycotic osteomyelitis of the maxilla, a very rare bacterial superinfection that occurred after an orofacial HZ infection. To our knowledge, this is the first reported case of actinomycotic osteomyelitis of the maxilla following an HZ infection.

## Case presentation

A 61-year-old man reported to the Department of Oral Medicine and Radiology with the chief complaint of multiple ulcers and swelling on the left side of the face for the past week. History revealed that he had no comorbidities and had undergone an extraction of the left upper back tooth 28, after which he developed rigors, fever, and vesicles, which later ruptured into ulcers on the left side of his face. The patient also reported a history of numbness and swelling associated with the ulcers. The patient consulted a physician and was prescribed antiviral drugs, steroids, and antihistamines. However, the patient had no symptomatic relief with the medications prescribed and reported an increase in the severity of disease presentation associated with burning pain when he visited the department.

**Figure 1:**
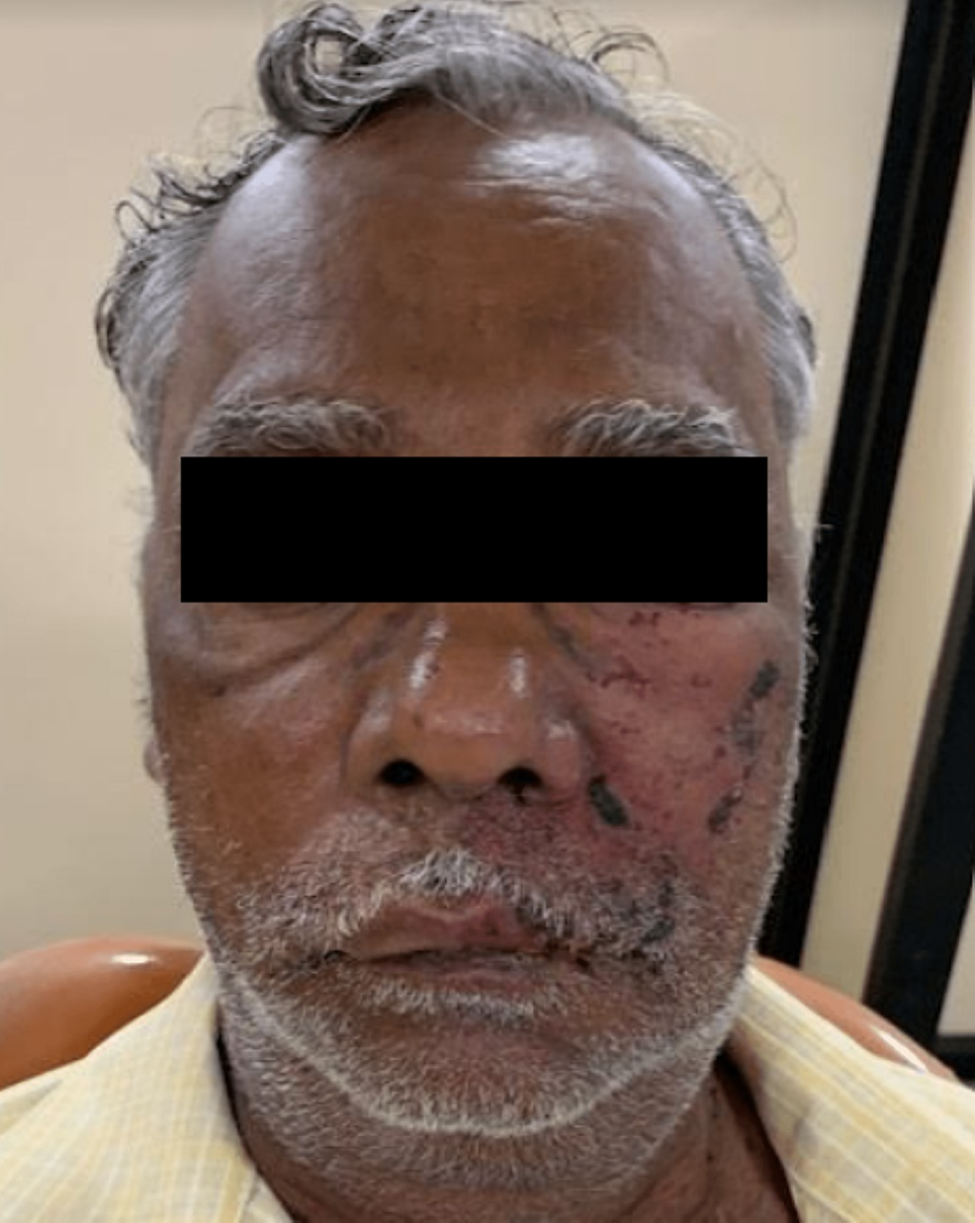
Extraoral swelling. Diffused extraoral swelling with scars limited to the left maxillary and mandibular division of the trigeminal nerve.

An extraoral examination revealed diffuse extraoral swelling involving the left middle-third of the face with obliteration of the nasolabial fold. Scars were evident on the left side of the face in relation to the middle and lower third of the face not crossing the midline. The scars were limited to the distribution of the left maxillary and mandibular divisions of the trigeminal nerve (Figure 1).

**Figure 2:**
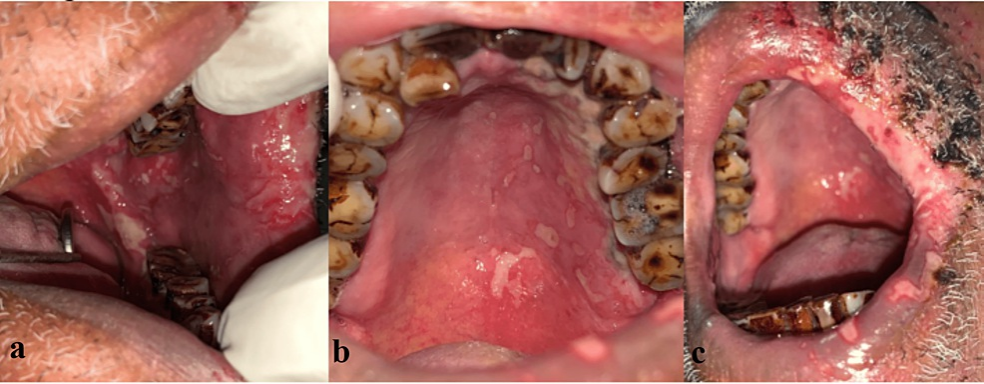
Multiple ulcers involving multiple areas of the oral cavity limited to the left trigeminal nerve distribution. a: Ulcers involving the left retromolar region and the buccal mucosa. b: Ulcers involving the left side of the hard palate without crossing the midline. c: Ulcers involving the left side of the upper and lower lip.

Intraoral evidence of multiple ulcers with erosions and irregular borders surrounded by erythema was found, which were limited to the left side involving the retromolar region, buccal mucosa, hard palate, and commissure of the lip (Figure 2). There was no evidence of bleeding. Paresthesia was elicited. Based on the patient’s history and clinical appearance, an HZ infection was provisionally diagnosed.

**Figure 3:**
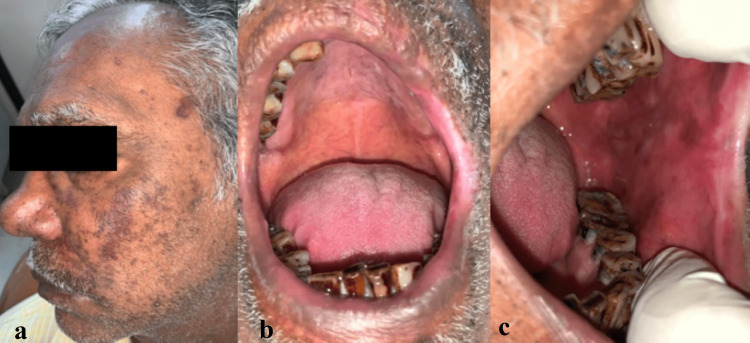
Post-treatment. a: Evidence of resolved scars with pigmentation. b: Evidence of resolved ulcers in the hard palate, upper lip, and lower lip. c: Evidence of resolved ulcers in the retromolar region and the buccal mucosa.

During the investigation, a salivary polymerase chain reaction test (PCR) revealed the presence of the varicella-zoster virus, and blood investigation results were within limits. Based on confirmatory investigations, the patient was treated for an HZ infection with tablet valacyclovir (1 g), methylcobalamin (750 µg), and pregabalin (75 mg) once daily and ointment mupirocin 2% and ointment acyclovir 1% thrice daily for one week. A follow-up revealed completely healed ulcers with persistent burning pain. The patient was asked to continue methylcobalamin (750 µg) and pregabalin (75 mg) once daily for one month, suspecting the case was leading to PHN. On his next visit after one week, the patient was completely asymptomatic with healed lesions (Figure 3).

**Figure 4:**
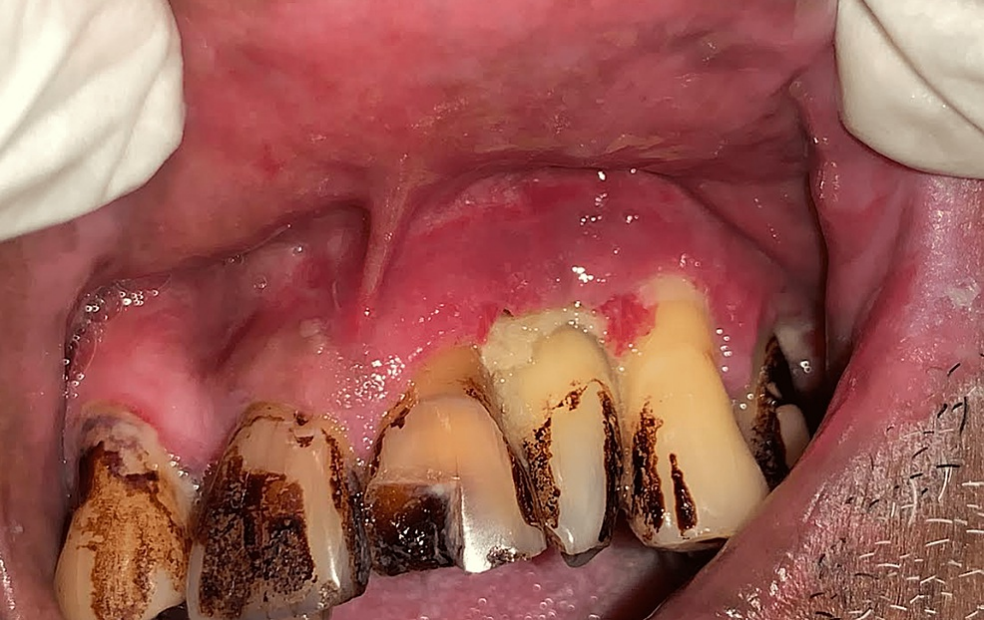
Evidence of gingival inflammation with pus discharge in relation to the 21, 22, 23 region.

However, he reported an additional complaint of bleeding and pus discharge from the gums with mobile teeth from 21 to 24 (Figure 4). Further history revealed nasal regurgitation on intake of oral fluids. The patient was subjected to a swab test to access microbial culture, which revealed a few gram-positive cocci along with pus cells in association with *Enterococcus faecalis*. On further investigation, a fungal culture test was negative, and an antibiotic sensitivity test revealed cephalexin and ciprofloxacin resistance.

**Figure 5:**
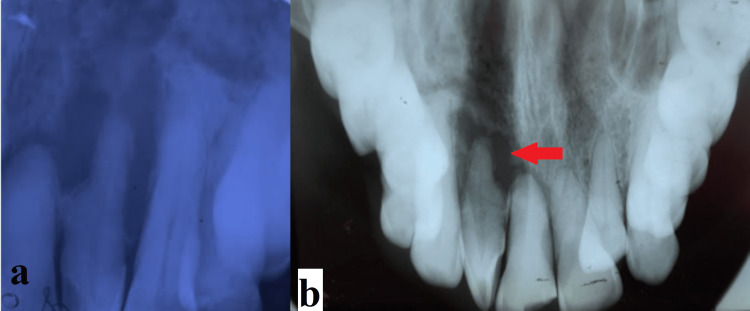
Intraoral periapical radiograph and occlusal radiograph. a: Intraoral periapical radiograph showing ill-defined periapical radiolucency with bone loss in relation to the 21, 22, 23 region. b: Occlusal radiograph revealing mixed radiopaque and radiolucent area superior to periapical radiolucency with altered trabeculae pattern in relation to the 21, 22, 23 region (red arrow).

An intraoral periapical radiograph in relation to 21, 22, 23, 24 revealed ill-defined periapical radiolucency with bone loss (Figure 5). Occlusal radiograph revealed mixed radiopaque and radiolucent area superior to periapical radiolucency with altered trabeculae pattern (Figure 5).

**Figure 6:**
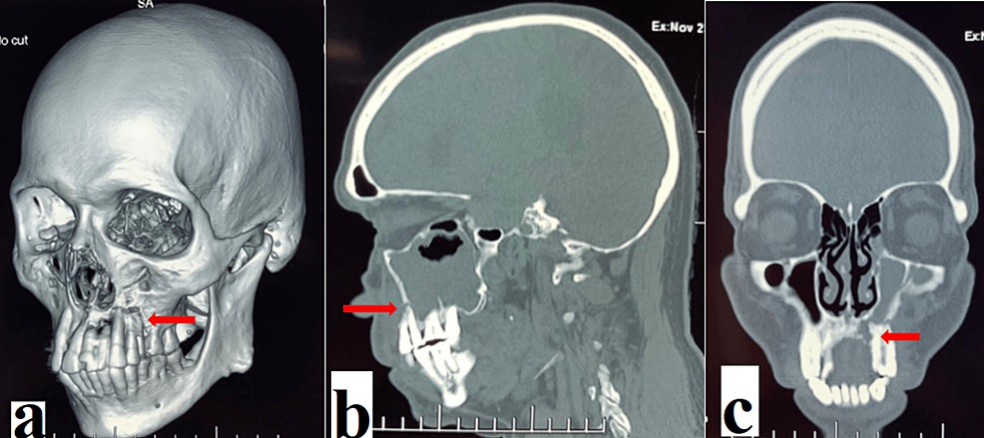
Computerized tomography of the paranasal sinuses with three-dimensional reconstruction. a: Three-dimensional reconstruction revealing bony erosion involving the left alveolar process. b: The sagittal view revealing bony erosion involving the left alveolar process and the hard palate involving the pre-molar region. c: The coronal view revealing fistula formation between the oral cavity and the maxillary sinus.

The patient was further subjected to computerized tomography of the paranasal sinus with three-dimensional reconstruction, which revealed bony erosion involving the left alveolar process and the hard palate involving the pre-molar region, resulting in fistula formation between the oral cavity and maxillary sinus, suggestive of a left oro-antral fistula (Figure 6).

**Figure 7:**
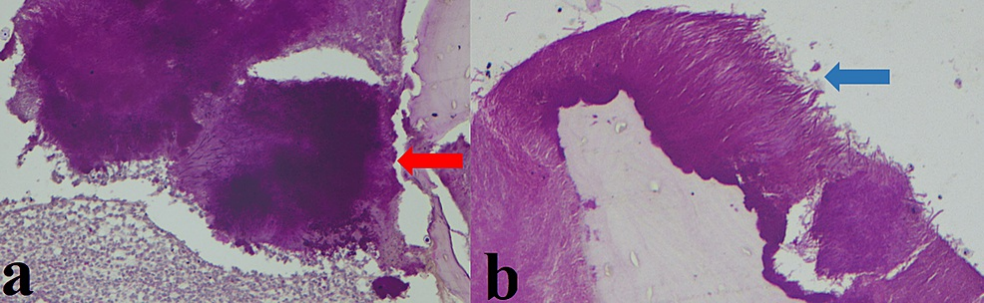
Histopathological picture. a: The section shows connective tissue stroma with mixed inflammatory cell infiltration predominantly lymphocytes, plasma cells, and neutrophils surrounding numerous areas of bacterial colonies along with Splendore–Hoeppli phenomenon (red arrow). b: The section shows basophilic radiating filaments (blue arrow).

An incisional biopsy performed following the extraction of 22, 23, 24 revealed peripheral stratified squamous epithelium and connective tissue stroma with mixed inflammatory cell infiltration, predominantly lymphocytes, plasma cells, and neutrophils surrounding numerous areas of bacterial colonies, basophilic radiating filaments along with the evidence of necrotic bones (Figure 7), proving to be actinomycosis osteomyelitis.

**Figure 8:**
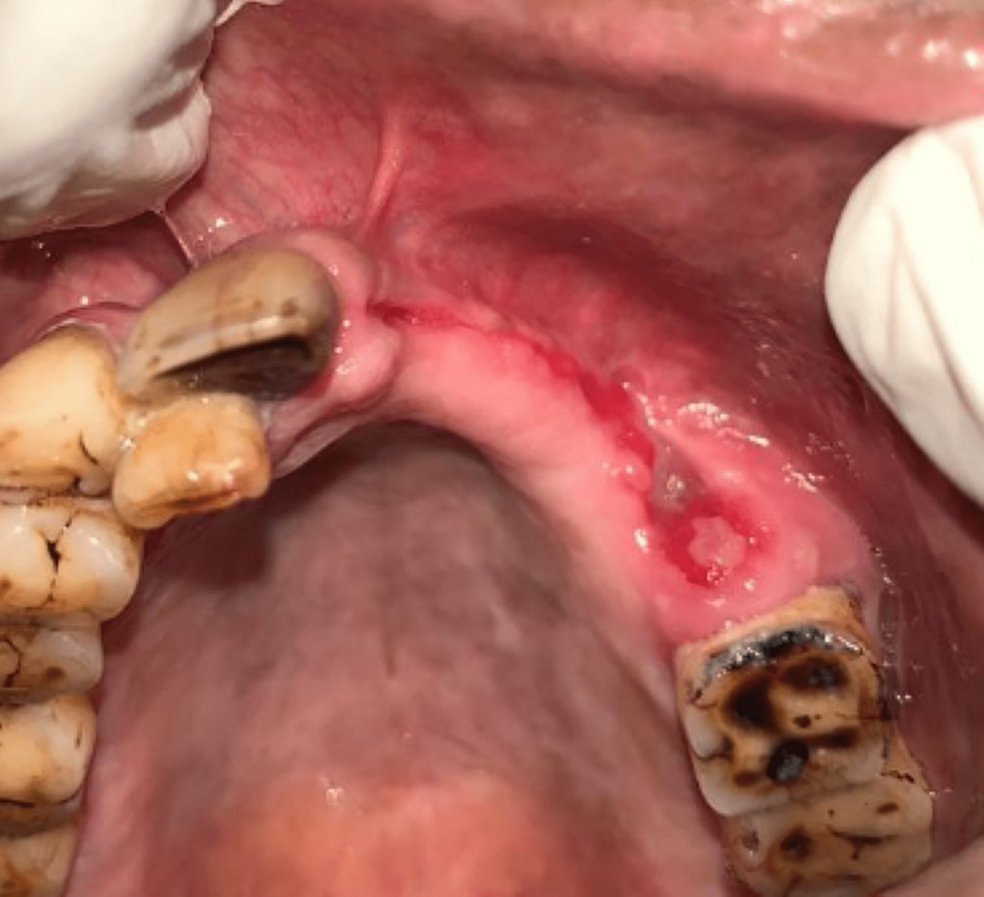
Intraoral image post-treatment with ceftriaxone 1 g.

Therefore, this case was a rare complication of post-HZ infection, leading to actinomycotic osteomyelitis on final diagnosis. Further, the patient was administered ceftriaxone 1 g intravenously for one week. On subsequent visits, the patient was symptomatically better, with no nasal regurgitation (Figure 8).

## Discussion

Maxillary and mandibular alveolar bone necrosis caused by an HZ infection is uncommon. Spontaneous tooth exfoliation and post-herpetic alveolar necrosis have been documented in 51 cases as of 2021 [5-12]. Gupta et al. reported 46 similar instances in a recent literature review, with a mean age of 52 years [5]. According to Gholami et al., bone necrosis often manifested as a serious infection between nine and 150 days following the beginning of shingles [6]. The average duration was 30 days. In the present case, the patient presented with an edematous gingiva with pus discharge following two weeks of shingles infection. To ensure the patient’s overall well-being, regular and prolonged follow-ups over several months may be recommended.

Gholami et al. reported the case of a 53-year-old woman with mandibular osteonecrosis after 28 days of shingles infection with microscopic features of osteomyelitis along with intertrabecular spaces filled by necrotic tissue and bacterial colonies [6]. In this paper, we reported a case of actinomycotic osteomyelitis with the classical feature of actinomycosis Splendore-Hoeppli reaction, radiating filamentous (Figure 7). Histologically, the bacilli are bordered by eosinophilic amorphous material with a club-shaped configuration known as the Splendore-Hoeppli reaction seen in Infective conditions such as actinomycosis, aspergillosis, blastomycosis, botryomycosis, and candidiasis [13]. To our knowledge, this is the first case to report actinomycotic osteomyelitis followed by shingles infection.

The anaerobic gram-positive bacterium *Actinomyces israelii* often causes actinomycosis, an uncommon kind of saprophytic bacterial illness. Factors including the disruption of the oral mucosa and/or systemic factors such as diabetes mellitus or other immunocompromised conditions, poor dental hygiene, a tooth infection, or trauma enhance the chances of an actinomycotic infection. The mandibular area is more commonly affected than the maxillary region due to extensive vascularity [14]. Although our patient did not have any history of trauma, due to poor oral hygiene, actinomycosis infection could have manifested. In the review of the English-language literature reported by Fazili et al., a significant number of patients with poor oral hygiene and a history of alcoholism were identified in a total of 32 cases of actinomycosis infection [15].

The other hypothesis is that the antiviral drug could lead to immunopathological death of antigen-presenting cells, macrophages, follicular dendritic cells, and some CD4+ T cells, resulting in general immunosuppression and reducing the immune system’s ability to respond to outside antigens. In addition, it also speeds up the exhaustion and deletion of CD8+ T-cell responses that are specific to the lymphocytic choriomeningitis virus [16,17]. Therefore, our patient, who was originally prescribed an antiviral medication to treat HZ, may have developed maxillary actinomycotic osteomyelitis as a result of the immune system-inhibiting effects of the medication.

There was a case of actinomycosis osteomyelitis following COVID-19 infection that involved invasive actinomycosis infection, including the unusual location of the maxilla. This case also featured a decreased lymphocyte count following COVID-19 infection. The administration of severe immunosuppression led to actinomycosis. Further local actinomycotic spread was facilitated by the inclusion of necrotic tissues [18].

Combining surgical and pharmacological therapy is a widely recognized therapeutic strategy, despite ongoing debate. Only after a long-term, strict antibiotic treatment has failed, surgery is performed. As infection may reappear after a period of inactivity, long-term follow-up is important [14,16,18]. In our case, the patient’s symptoms were relieved with ceftriaxone 1 g intravenous medication for one week, and the patient was advised o undergo regular follow-ups.

## Conclusions

We summarize that actinomycotic osteomyelitis may be brought on by HZ as a complication. Maxillary osteomyelitis is a relatively uncommon illness, and in this instance, actinomyces was the etiological agent, making it much less likely. Actinomycotic infection should be ruled out in patients who have had HZ because it may be the only, or a significant, reason for their recurrent or chronic oral infections. To ensure patients’ well-being, actinomycosis as a potential complication of HZ must be kept in mind during diagnosis.
